# Quantum dot single-photon switches of resonant tunneling current for discriminating-photon-number detection

**DOI:** 10.1038/srep09389

**Published:** 2015-03-23

**Authors:** Qianchun Weng, Zhenghua An, Bo Zhang, Pingping Chen, Xiaoshuang Chen, Ziqiang Zhu, Wei Lu

**Affiliations:** 1 National Laboratory for Infrared Physics, Shanghai Institute of Technical Physics, Chinese Academy of Sciences, Shanghai 200083, PR China; 2 Key Laboratory of Polar Materials and Devices, Ministry of Education, East China Normal University, Shanghai 200241, PR China; 3 State Key Laboratory of Surface Physics, Key Laboratory of Micro and Nano Photonic Structures (Ministry of Education) and Institute of Advanced Materials, Fudan University, Shanghai 200433, PR China; 4 Collaborative Innovation Center of Advanced Microstructures, Fudan University, Shanghai 200433, PR China

## Abstract

Low-noise single-photon detectors that can resolve photon numbers are used to monitor the operation of quantum gates in linear-optical quantum computation. Exactly 0, 1 or 2 photons registered in a detector should be distinguished especially in long-distance quantum communication and quantum computation. Here we demonstrate a photon-number-resolving detector based on quantum dot coupled resonant tunneling diodes (QD-cRTD). Individual quantum-dots (QDs) coupled closely with adjacent quantum well (QW) of resonant tunneling diode operate as photon-gated switches- which turn on (off) the RTD tunneling current when they trap photon-generated holes (recombine with injected electrons). Proposed electron-injecting operation fills electrons into coupled QDs which turn “photon-switches” to “OFF” state and make the detector ready for multiple-photons detection. With proper decision regions defined, 1-photon and 2-photon states are resolved in 4.2 K with excellent propabilities of accuracy of 90% and 98% respectively. Further, by identifying step-like photon responses, the photon-number-resolving capability is sustained to 77 K, making the detector a promising candidate for advanced quantum information applications where photon-number-states should be accurately distinguished.

Driven by explosive growth of the field of quantum-information science^1^,^2^,^3^,^4^,^5^ over the last few decades, new single photon detector^6^,^7^,^8^,^9^,^10^,^11^ technologies have attracted dramatic interest. Most conventional single-photon detectors have a binary response, which can only distinguish between “zero” or “more than zero photons”, and multi-photons induce the same output signal as single photon. However, pushed by the urgent application demands from quantum computation, quantum teleportation^12^ and characterization of quantum light sources^13^, expanding the photon-counting capability of existing single-photon detectors to photon-number-resolving are highly anticipated^6^,^14^,^15^. Recently an efficient single-photon detector based on resonant tunneling diodes is presented by the pioneer work of Blakesley *et al*^16^. The resonant tunneling process is affected by single charge in nanoscale quantum dot via its electrostatic potential. With considerable effort taken for the improvements^17^,^18^,^19^, this device is identified to measure single photons with extremely low dark count rates (<10^−2^ Hz) and therefore the best reported figures of merit (FOM)^20^. However, up to now no photon-number resolving ability of this RTD-based detector has ever been realized, which is although mandatory for many quantum information applications. In this work, we introduce QD coupled resonant tunneling diodes (QD-cRTD), in which the QW of resonant tunneling structure and nearby negatively charged QD are spaced by a very thin barrier allowing not only efficient capacitive coupling like Blakesley's work^16^ but also quantum tunnel coupling. Eventually the resonant current can be effectively switched on/off by incident photons. In addition, the appropriate electron-injecting operation manipulates coupled QD charge states and makes the device practicable for photon-number-discriminating detection. Excellent PNR performance is realized not only at a temperature of 4.2 K but also at 77 K.

## Results

**Figure 1:**
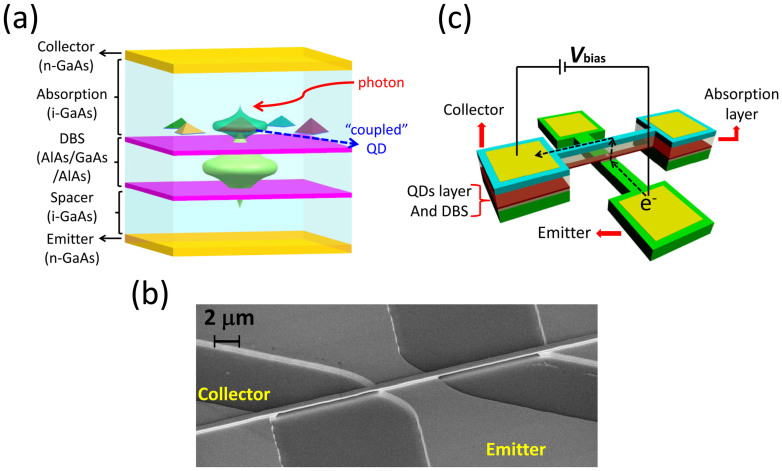
(a) Semiconductor layers (from bottom contact to top contact): a graded n-doped (doping level: from 1 × 10^18^ cm^−3^ to 1 × 10^16^ cm^−3^) GaAs layer (430 nm) as bottom contact (or emitter), an intrinsic GaAs spacer layer (20 nm), an AlAs barrier layer (3 nm), a GaAs quantum well layer (8 nm), an AlAs barrier (3 nm), an intrinsic GaAs spacer layer (2 nm), a self-assembled InAs quantum dots layer capped by 10 nm GaAs, an intrinsic GaAs photon absorption layer (150 nm), and a n-doped (doping level: 2 × 10^18^ cm^−3^) GaAs layer (50 nm) as top contact (or collector). (b) Scanning electron microscope image of a cross-wire freestanding bridge. (c) Electron path from bottom contact (emitter) to top contact (collector) under positive bias for single-photon detection.

Figure 1(a) shows the basic composition of a quantum dot double barrier structure (DBS) used in the experiments. The thickness of resonant tunneling barrier is only 2 nm, therefore the electron wave function in the DBS quantum well (QW) is not fully confined by the barriers and can tail to the QD layer due to small barrier thickness (2 nm) between QW and QD. Meanwhile, some QDs are negatively charged by the proposed electron-injecting operation, which significantly pushing up the excited level of such a QD, making it closer to the confined state of the QW, therefore the coupling between QDs and QW occurs. The current flowing through the device can be divided into two parts: one is the current path with only DBS and no QD exist, which is not sensitive to the incident photons and only contributes to the dark current; the other type is the current paths with both DBS and QD, which are distributed individually and in parallel, depending on the location of nanoscale QD in the plane. When a QD is negatively charged, the potential of the QD is built up in conduction band and no electrons from emitter can tunnel through this DBS-QD nano-path, which means this nano-channel is closed, referred as “OFF” state. A photo-generated hole drifts toward the negatively charge QDs under applied electric field and neutralizes the electron stored there, causing observable resonant tunneling current through this nano-channel, namely this QD is switched to “ON” state by an incident photon. In this detection process, each negatively charged QDs operate as individual “single-photon switches” in parallel and the device can have the ability to respond to multiple-photons.

A cross-wire geometry^21^,^22^ is fabricated by standard photolithography and selective etching process for forming micro-nano active region. The top contact (collector) was well separated from the bottom contact (emitter) with crossed wires etched. After the fabrication process, a 200 nm thick and 600 nm wide freestanding bridge was formed by etching away the highly doped GaAs bottom contact layer under the top contact as shown in the scanning electron microscope (SEM) image of Figure 1(b), and only the crossed part was kept. Under positive bias as displayed in Figure 1(c), the only possible resonant tunneling region for an electron from bottom contact to the top contact is the intersection of the cross wires (~5 × 0.6 μm^2^). About 100~300 QDs are buried in the small active region by a rough estimation.

**Figure 2:**
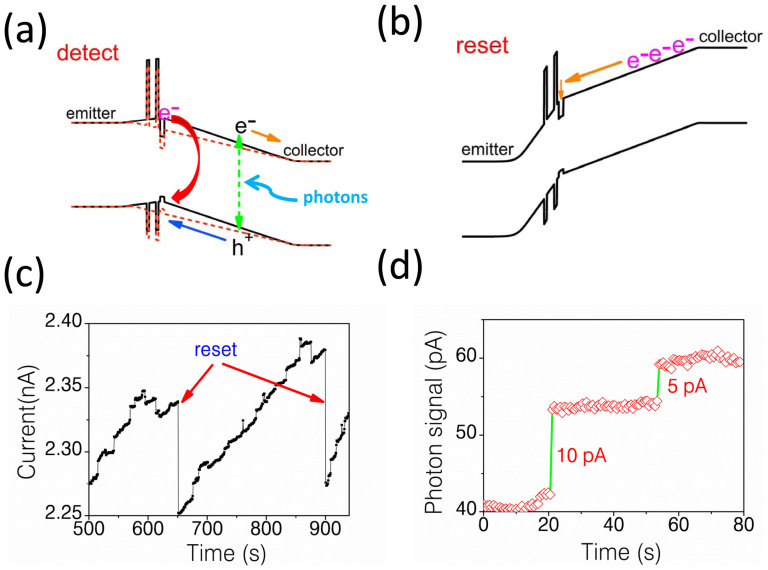
(a) Band diagrams of QD-cRTD when positively biased for single-photon measurements before (dark line) and after (red line) absorption photons. (b) Band diagram of QD-cRTD when negatively biased for efficient electron-injecting operation. (c) Time trace of single-photon detection with proposed electron-injecting operation. (d) Quantized step-like current signal due to different photon-number states detected.

Figure 2(a) and (b) shows the band diagram under forward bias for efficient single-photon detection (band conditions before and after absorption photons are shown in Fig. 2(a)) and under negative bias for electron-injecting operation separately. The pre-charging of QDs with negative pulse turn the QDs to “OFF” state and activate the QDs as workable “single-photon switches”. The key point of successfully injecting electrons into the QDs is the amplitude of reset pulse, rather than the amplitude of negative current flowing through QDs^23^.

Time-resolved photon measurements with periodical reset (electron-injecting) operation were carried out in 4.2 K when biasing the detector at 0.56 V and the result is displayed in Figure 2(c). A blue LED (~470 nm) is attenuated and used as the illumination source. Under weak illumination, the current increases and drops to initial value when each electron-injecting pulse applied. Notice that there is slope-like current increase together with large step-like current increase when the device is illuminated. Considering the small gap (~2 nm) between QD and DBS, the wave function of electrons in QD and QW overlaps spatially, and the excited level of QD was built-up by injected electrons which approaches the confined state in the QW, causing quantum coupling between QDs (0-dimension) and QW (2-dimensions). We attribute the large step-like current increase to photo-generated holes trapped in such kind of coupled negatively QDs. And other un-coupled QDs contribute to the slope-like current increase. The slope-like current contribution contaminates the current changes caused by coupled QDs and is the main obstacle to clearly distinguish between 0-photon and 1-photon as will be discussed later.

**Figure 3:**
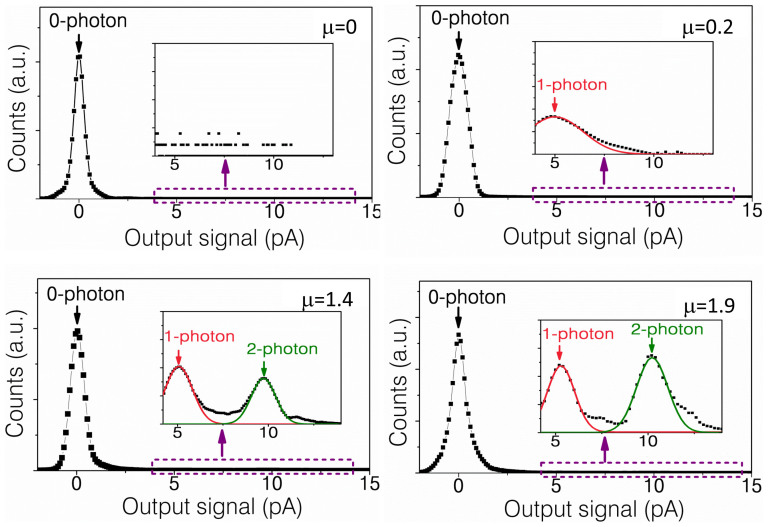
Photon-number-discrimination detection under different illumination levels in 4.2 K.

With plenty of electrons injected in the coupled QDs, the ability of the device to resolve the number of detected photons was demonstrated and investigated. Figure 2(d) gives clear quantized step-like photon signals which correspond to 1 photon and 2 photons detected. Further statistical photon measurements with periodically electron-injecting operation were carried out under different illumination levels and the results are plotted in Figure 3. The traces show a series of maxima which are centered around 0.0 pA, 5.0 pA and 10.0 pA, which we ascribe to the tunneling current induced by 0, 1 or 2 photons. In the absence of light, only single peak exists which associated with the detection of zero photons. The broaden of this distribution should be attributed to two parts: first, the electrical noise induced current fluctuations which is typically within 0.7 pA in our experiments; And second, the slope-like background current contributions by un-coupled QDs, which can spread to larger current level depending on illumination intensities and contaminate the step-like current induced by single-photon detection. By comparison, when weakly illuminating the detector, additional distinct peaks centered around 5.0 pA and 10.0 pA are observed, which are associated with the detection of 1 and 2 photons, respectively. Mainly due to the seriously degraded detection efficiency, the resolution of higher photon number states (≥3) is not clear in this detector.

With a perfect photon-number-resolving detector, the photon number states should be assigned to quite precise outputs of the detector with 100% probability of accuracy. However, practical detectors show some uncertain in response, setting decision regions^7^ for different photon number states would be a proper solution. In first row of Table 1 we list the estimated probability of correctly determining the photon number states in 4.2 K for defined decision regions. From the experimental data and Gaussian fits presented in Fig. 3, the boundaries of decision region for each photon number states are defined. Although there's no intersection between Gaussian functions of 1 photon and 2 photon numbers, which indicate that we can identify 1 photon and 2 photons with 100% accuracy in principle. However, non-uniformity of coupled-QDs cannot be completely avoided and can cause some uncertainty between 1 photon and 2 photons signals. Even taking it into considerations, we can still determine 2 photons with an extremely excellent accuracy larger than 98%, which is already better than the other QD based photon-number-resolving detector using field-effect amplification^7^. As discussed above, the existence of slope-like background current causes some difficulties to identify 0 photon and 1 photon. As a result, comparing to the 2 photon numbers detection, the probability of correctly determining the 1 photon number state is decreased, but still better than 90%.

**Figure 4:**
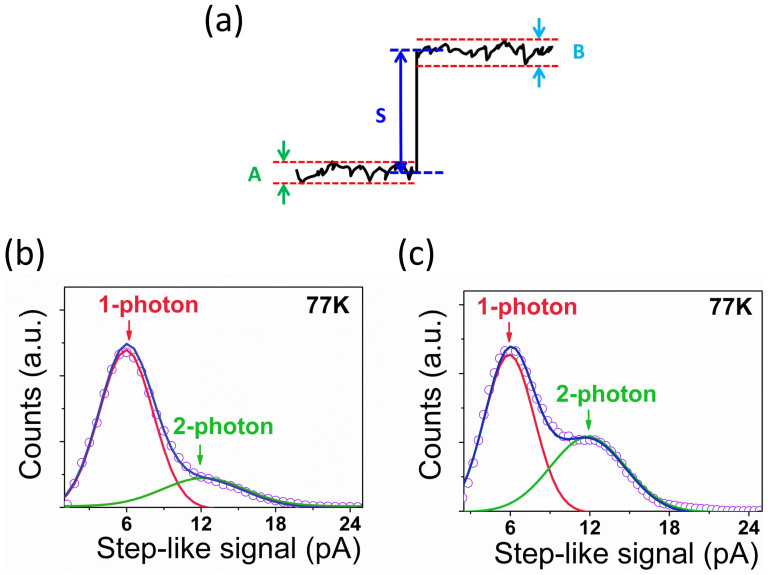
(a) Diagram of the method to identify the step-like photon signal against noise. (b), (c) Photon-number-discrimination detection in 77 K with the method of identifying photon signals with step-like feature under different illumination levels.

To resolve photon numbers in a relative high operation temperature (77 K) can make this photon-number-resolving detector more feasible for quantum information applications. Notice that incident photons induce quantized step-like response of the detector, by identifying the step-like feature of photon signals in real-time measurement (as shown in Figure 4(a), we set the rule that if the noise level A and B is smaller than 10% of the step-like signal strength S, then we note the signal S as the photons detected, otherwise we note it as noise), we demonstrate that the detector can resolve photon numbers even at 77 K. We bias the detector at the same voltage (0.56 V) as in 4.2 K and measure the photon numbers in 77 K. With the slope-like background current and electric noise suppressed by introduced identification method, quite clear 1 photon and 2 photon signals are resolved, in which the peaks are centered around 6.0 pA and 12.0 pA (as shown in Fig. 4(b) and (c)). The probabilty of accuracy when determining the photon number states is also estimated and is shown in second row of Table 1, which is still better than 85%. Totally, this QD-cRTD photon number resolving detector is a desirable candidate in applications where one or two photons register in the detector should be accurately determined^4^,^6^.

## Conclusion

We have demonstrated the photon-number-resolving capabilities of a semiconductor detector that uses self-assembled QDs which is coupled with QW of DBS as single-photon sensitive switches to open (or close) parallel individual nano-channels of resonant tunneling current. Well-defined peaks in histograms of the detector outputs correspond to the discrete photon numbers. The detector shows excellent photon number resolving ability both in 4.2 K and 77 K, but the quantum efficiency for photon-number-resolving detection is degraded seriously (<1%) compared to previous work in Ref. 16 due to existence of un-coupled QDs which contributes to the slope-like current increase even under weak illuminations. Further studies will include the better control of coupling between QDs and QW. If stronger coupling is introduced, single-photon induced step-like signal will be significantly enhanced for high temperature photon number detection and slope-like background current can be negligible small in that case.

## Methods

The photon-number-discriminating detection by the quantum dot double-barrier structure is studied at 4.2 K and 77 K by illuminating the detector with an attenuated LED and measuring the weak current change through double-barrier structure in real-time. Periodical electron-injecting operation is applied with a frequency of 0.004 Hz, which reset the coupled QDs to fully negatively charged state for photon-number detection and avoid the detector approaching to saturation due to slope-like background current. The amplitude of electron-injecting pulse should be large enough to distort conduction band profile for efficiently inject electrons into the QDs. In 4.2 K, when biased the detector at 0.56 V, the single-photon induced signal is 5.0 pA, whereas in 77 K at the same bias the single-photon signal is 6.0 pA, which led to different decision-region boundaries in those two situations. For the photon number detection measurements in 4.2 K, the detector is placed at the bottom of a long sample holder which is immersed in liquid helium. The illumination is attenuated at room temperature and guided through a thin waveguide towards the detector. As for the measurements in 77 K, a dewar is used to keep the detector and cooled to 77 K. The illumination is attenuated and directly shine on the detector. In order to estimate the mean incident photon numbers and therefore the mean detected photon numbers, we calibrated the transmission efficiency of the optical setup in the following way. We measure the photon intensities with a Si detector both at the LED source position and at the end of optical path (that is the device position where real photon detection measurements were performed). The ratio between two intensities gives the transmission value of our optics, which is ~0.6% for 77 K measurement and ~0.12% for 4.2 K. When the LED drive current is small, the LED emission intensity changes linearly with the drive current, therefore the mean photon number reaching the detector can be linearly tuned by changing the LED current and evaluated directly from the LED emission intensity. For the case of mean detected photon number ~1.4, the LED emission intensity was measured by the Si detector at the source position to be 1.4 nW (or ~3.31 × 10^9^ photons/sec). So the photon flux reaching the active region of detector is approximately 3.31 × 10^9^ photons/sec × 0.12% × 4.3 × 10^−5^ = 171 photons/sec, in which 4.3 × 10^−5^ is the ratio of device active region(~3 μm^2^) and the optical sensitive area of Si detector (diameter = 0.3 mm, active area ~0.07 mm^2^). The quantum efficiency is estimated to be ~0.8%, therefore the mean detected photon numbers of 1.4 is derived and estimated.

## Author Contributions

Q.C.W. performed the device fabrication and all the measurements. Z.H.A., B.Z., X.S.C. and Q.C.W. performed the data analysis. P.P.C. grew the wafer. Z.Q.Z. and W.L. supervised the project. Q.C.W. and Z.H.A. contribute equally to this work.

## Figures and Tables

**Table 1:** Probabilities of correctly determining the photon number states

Temperature	Photon number	Decision region	Percent correct
**4.2 K**	**0**	*I_step_* ≤ 4.0 *pA*	**~100**
	**1**	4.0 *pA* < *I_step_* ≤ 7.5 *pA*	**90**
	**2**	7.5 *pA* < *I_step_* ≤ 14.0 *pA*	**98**
**77 K**	**0**	**No step-like feature**	**~100**
	**1**	2.80 *pA* < *I_step_* ≤ 8.89 *pA*	**89**
	**2**	8.89 *pA* < *I_step_* ≤ 21.0 *pA*	**85**
